# Comparative Evaluation of Combinatory Interaction between Endocannabinoid System Compounds and Poly-L-lysine against *Streptococcus mutans* Growth and Biofilm Formation

**DOI:** 10.1155/2020/7258380

**Published:** 2020-01-31

**Authors:** Mark Feldman, Ronit Sionov, Reem Smoum, Raphael Mechoulam, Isaac Ginsburg, Doron Steinberg

**Affiliations:** ^1^Biofilm Research Laboratory, Faculty of Dental Medicine, The Hebrew University of Jerusalem, Jerusalem, Israel; ^2^The Institute for Drug Research, School of Pharmacy, The Hebrew University of Jerusalem, Jerusalem, Israel

## Abstract

Endocannabinoid/endocannabinoid-like (EC/EC-like) are natural endogenous compounds which have been found to affect MRSA pathogenicity. Our previous studies showed that EC/EC-like was able to impair staphylococcal biofilm formation and maintenance as well as to alter biofilm-associated virulence factors. In the present study, we investigated the combinatory effect of the selected EC/EC-like with a natural antimicrobial agent, poly-L-lysine, on cariogenic bacteria *Streptococcus mutans* growth and biofilm formation. Among four tested EC/EC-like, only two, anandamide (AEA) and oleoylethanolamide (OEA), exhibited synergistic combinatory effect with poly-L-lysine against *S*. *mutans*. We attribute this distinct effect to differences in the fatty acid chain structure of the selected EC/EC-like compounds. Moreover, AEA exerted a specific antibiofilm mode of action against *S*. *mutans* by effecting total inhibition of biofilm formation while still allowing bacteria viability. Finally, we postulate that the presence of EC/EC-like and poly-L-lysine could enhance the permeability and efficacy of each other via hydrophobic and electrostatic interactions with the *S*. *mutans* membrane. In conclusion, we assume that a combination of endogenous natural compounds such as EC/EC-like and poly-L-lysine may benefit oral hygiene by preventing dental plaque.

## 1. Introduction

The oral cavity is the primary entry point for nutrients and fluid and it contains saliva produced by glands. Many microbial species are found in saliva that may grow in planktonic form but also from dense biofilms. The latter may accumulate on either teeth, inflamed gingivae, or also orthodontic appliances. Such biofilms are highly impervious to antimicrobial agents and may also invade the bloodstream to cause sepsis and septic shock. *Streptococcus mutans* are among the prominent bacterial species found in dental plaque.

Effective control of oral bacteria can be achieved by using multiple antimicrobials that provide synergistic action of the combined substances against targeted microorganisms. For instance, it has been shown that a combination of a hepatoprotective agent, silibinin, and antibiotics synergistically inhibited growth of a wide range of oral bacteria [1]. Another study demonstrated the synergistic effect of essential oils and tetracycline against growth and biofilm formation of oral pathogens [2]. Recently, a variety of combinatory anticariogenic approaches have been applied with respect to *S*. *mutans* [3–6].

Poly-L-lysine is a homopolymer of 25–30 L-lysine residues naturally produced by *Streptomyces albulus* subsp. lysinopolymerus and is safe for human consumption [7]. It has demonstrated no toxicity to mammalian cells in animal studies with high doses [8]. In addition, this agent was shown to be stable within a broad spectrum of temperature and pH [8]. Poly-L-lysine is a cationic, surface active agent due to its positively charged amino groups and is therefore associated with strong antimicrobial activity against a wide range of microorganisms [9, 10]. Poly-L-lysine was able to damage *Escherichia coli* cell membranes [11]. This polycation efficiently kills *Pseudomonas aeruginosa* and *Staphylococcus aureus* in cystic fibrosis (CF) sputum and, therefore, could be administrated as an alternative to classical antibiotics in CF [12]. Furthermore, poly-L-lysine acts synergistically with different compounds against a variety of pathogens: food-borne [13, 14], vaginal [15, 16], and oral including *S*. *mutans* [17].

The endocannabinoid system (ECS) is composed of endocannabinoids (ECs) and enzymes responsible for the synthesis and degradation of the EC, as well as CB1 and CB2, which are the cannabinoid receptors widely distributed throughout the body. These cannabinoid receptors are activated by different ligands, either endogenous, such as EC, or exogenous, such as plant cannabinoids as well as synthetic compounds [18]. The ECS is associated with the regulation of several human physiological processes, such as sleep and the immune response. Anandamide or N-arachidonoylethanolamine (AEA) is one of the main endogenous natural ligands of the cannabinoid receptors. It is recruited during tissue injury to provide a first response to nociceptive signals [19, 20]. The ECS also includes other EC-like compounds such as N-acylethanolamines (NAEs): oleoylethanolamide (OEA), palmitoylethanolamide (PEA), and stearoylethanolamide (SEA), whose molecular targets do not include CB receptors [21]. Similarly to EC, NAEs affect pain and inflammation [22]. There are limited data in the literature on antimicrobial activity of ECS-related compounds. Recently, we demonstrated that the AEA and EC-like AraS compound effectively alter the pathogenicity of different MRSA strains [23]. These agents notably inhibited biofilm formation and eradicated matured biofilms without affecting bacterial viability [23]. Staphylococcal biofilm-associated virulence determinants such as hydrophobicity, cell aggregation, and spreading ability were strongly altered by AEA and AraS [23]. We postulated that the mechanism of anti-MRSA nonbactericidal action of AEA and AraS is related to the modification of bacterial membrane and subsequent alteration of biofilm-associated properties of MRSA [23]. In addition, we found that the combination of the above agents and certain antibiotics synergistically altered MRSA biofilm formation, biofilm structure, and slime production (unpublished data). Finally, AEA has been demonstrated to attenuate virulence factors of *V*. *harveyi*, such as quorum sensing and motility [24].

In the present study, we describe the combinatory effect of cationic poly-L-lysine and EC/EC-like on planktonic growth and biofilm formation of *S*. *mutans*.

## 2. Materials and Methods

### 2.1. The Tested Compounds

**Figure 1:**
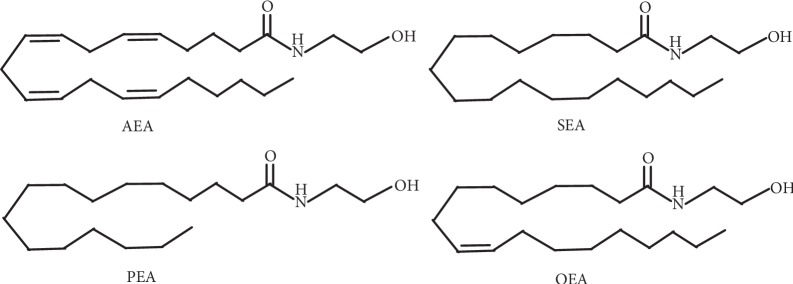
Structure of EC/EC-like.

AEA, OEA, PEA, and SEA (Figure 1) were synthesized in our laboratory following the chemical procedure described by Sheskin et al. [25]. Poly-L-lysine (MW 4–15 kDa) was purchased from Sigma (Sigma-Aldrich, St. Louis, MO).

### 2.2. Bacteria

*S*. *mutans* UA159 was incubated in Brain Heart Infusion Broth (BHI, Difco Labs, Detroit, USA) at 37°C in 5% CO_2_. Cultures of *S*. *mutans* were diluted 1 : 50, inoculated into fresh BHI media, and grown in polystyrene tubes for 24 h for planktonic culture generation.

### 2.3. Combinatory Effect of EC/EC-Like and Poly-L-lysine in Planktonic Condition

The stock solutions and serial twofold dilutions of each of the tested compounds (ECs) and of poly-L-lysine were prepared according to the recommendations of NCCLS immediately prior to testing. Briefly, each EC was serially diluted along the ordinate side of the 96-well microdilution plate, while poly-L-lysine was diluted along the abscissa. The range of final concentration of tested agents was 1.56  *μ*g/ml–25  *μ*g/ml for poly-L-lysine and 3.6  *μ*g/ml–25  *μ*g/ml for EC/EC-like. Bacterial inoculum of 0.5 McFarland turbidity was prepared from *S*. *mutans* UA159 strain. The resulting checkerboard is multiple combinations of the two agents. The 96-well plates were then incubated at 37°C for 24 h and supernatants from each well were transferred to the new 96-well plate. Planktonic growth was quantified by measuring the absorbance at 595 nm using a spectrophotometer (Genius Plate Reader, Tecan, Salzburg, Austria). The data are presented as a percentage of *S*. *mutans* growth and compared to an untreated control sample (100%) [23].

### 2.4. Combinatory Effect of EC/EC-Like and Poly-L-lysine in Biofilm

#### 2.4.1. Checkerboard Assay for Biofilm

The assay was performed under similar conditions to those described above with the addition of 2% sucrose (final concentration) to the growth media in order to allow sucrose-dependent biofilm formation of *S*. *mutans*. After incubation for 24 h, supernatant fluid was removed by aspiration and the wells were carefully washed twice with phosphate-buffered saline (PBS, pH 7.4). The biofilm was measured by crystal violet staining [23]. Briefly, 0.02% crystal violet solution was placed on top of the biofilm for 45 min, which was then washed twice with DDW to remove unbound dye. The dye was quantified by extraction with acetic acid. After adding 200 *μ*l of 30% acetic acid into each well, the plate was shaken for 10 min to release the dye and the biofilm was quantified by measuring the absorbance of the extracted dye at 595 nm using a spectrophotometer (Genius Plate Reader, Tecan, Salzburg, Austria). The data are presented as a percentage of *S*. *mutans* biofilm formation and compared to an untreated control (100%).

### 2.5. Growth/Biofilm Ratio

In order to evaluate the mode of antibacterial action for the combination of EC/EC-like with poly-L-lysine, we calculated the planktonic growth/biofilm formation ratio. A high ratio indicates a more antibiofilm-specific effect of the tested agents.

### 2.6. Statistical Analysis

The data represent the means ± SD of three independent experiments. The statistical analysis was performed on either single or combined treatment using Student's *t*-test with a significance level of *P* < 0.01 as compared to untreated controls.

## 3. Results

### 3.1. Combinatory Effect of the Compounds in Planktonic Condition

**Figure 2:**
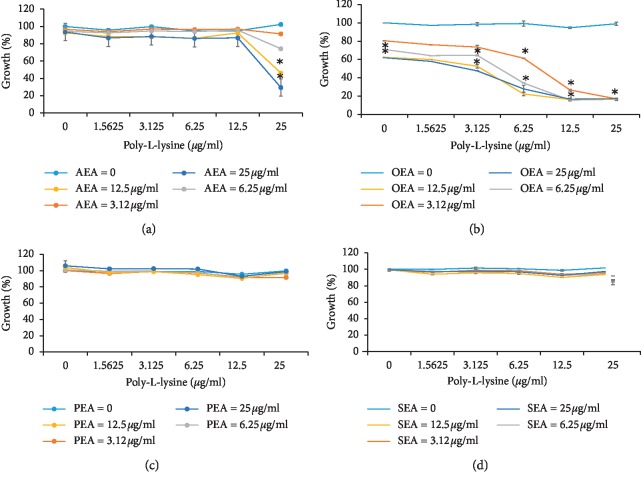
The combinatory effect of poly-L-lysine and EC/EC-like on *S*. *mutans* planktonic growth. The data represent the average of three independent experiments. ^*∗*^Significantly lower than the value for the untreated control, *P* < 0.01.

None of the agents showed minimal inhibitory concentration (MIC) at all tested doses (Figure 2), although a notable reduction in bacterial growth was observed at higher concentrations of the two compounds combined. In addition, poly-L-lysine applied alone did not decrease bacterial growth (Figures 2(a)–2(d)). Two tested EC-like, PEA and SEA, had no effect on *S*. *mutans* growth either alone or in combination with poly-L-lysine (Figures 2(c) and 2(d)). In contrast, the combination of either AEA or OEA with poly-L-lysine notably reduced bacterial growth (Figures 2(a) and 2(b)). Both AEA alone and AEA mixed with poly-L-lysine at doses up to 12.5 *μ*g/ml had no effect on bacterial growth (Figure 2(a)). However, an increase in the poly-L-lysine dose to 25 *μ*g/ml significantly enhanced the inhibitory pattern of the compounds' combination. When bacteria were exposed to mixture of the highest concentration of poly-L-lysine of 25 *μ*g/ml and AEA at doses of 6.25 *μ*g/ml, 12.5 *μ*g/ml, or 25 *μ*g/ml, the growth was notably and dose-dependently decreased by 26%, 54%, and 71%, respectively, as compared to the untreated control (Figure 2(a)). Even more pronounced combinatory inhibition was demonstrated by a mixture of poly-L-lysine and OEA. OEA alone was able to moderately reduce bacterial growth at doses of 12.5 *μ*g/ml and 25 *μ*g/ml by 40% (Figure 2(b)) as compared to the untreated control. However, growth of *S*. *mutans* was dramatically reduced by 80–86% when bacteria were incubated with a mixture of poly-L-lysine at doses of 6.25 *μ*g/ml–25 *μ*g/ml and OEA at doses of 6.25 *μ*g/ml–25 *μ*g/ml (Figure 2(b)).

### 3.2. Combinatory Effect of the Compounds in Biofilm

**Figure 3:**
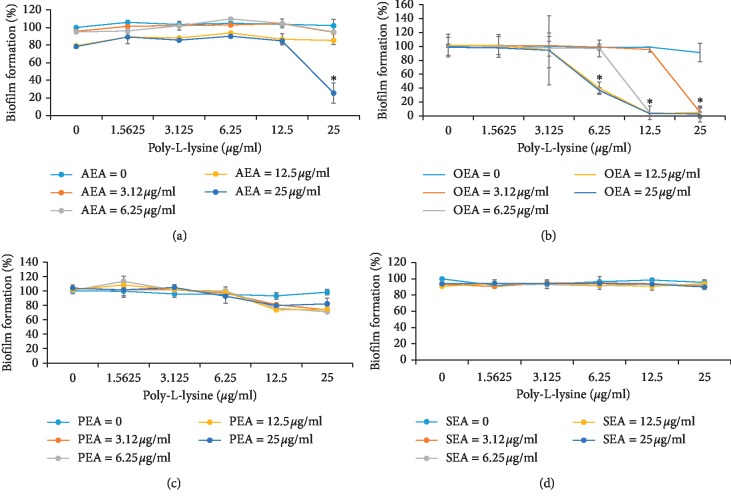
The combinatory effect of poly-L-lysine and EC/EC-like on *S*. *mutans* biofilm formation. The data represent the average of three independent experiments. ^*∗*^Significantly lower than the value for the untreated control, *P* < 0.01.

None of the tested compounds used alone had a statistically significant inhibitory effect on biofilm formation (Figures 3(a)–3(d)). In addition, the combination of either PEA (Figure 3(c)) or SEA (Figure 3(d)) with poly-L-lysine did not affect biofilm formation in any of the tested doses. In contrast, AEA or OEA in combination with poly-L-lysine remarkably decreased *S*. *mutans* biomass (Figure 3(a) and 3(b)). AEA alone at concentrations of 12.5 *μ*g/ml and 25 *μ*g/ml was able to reduce biofilm formation by 20% only. On the other hand, a mixture of AEA and poly-L-lysine at a dose of 25 *μ*g/ml dramatically reduced biofilm formation by 75% (Figure 3(a)). The most effective EC-like, OEA, in all tested doses with the addition of poly-L-lysine at 12.5 *μ*g/ml and 25 *μ*g/ml almost completely diminished *S*. *mutans* biofilm formation (Figure 3(b)).

### 3.3. Calculation of the Planktonic Growth/Biofilm Formation Ratio

The next step was to calculate planktonic growth/biofilm formation ratios in order to determine the specific antibiofilm effect of the tested combinations. The majority of growth/biofilm ratios with regard to the combinatory effect of AEA, PEA, and SEA with poly-L-lysine were close to 1 (Table 1 (A, C, and D)). Based on these data, we can conclude the following: the inhibitory effect of AEA in combination with poly-L-lysine on biofilm formation is due to the reduction in bacterial growth and was defined as nonspecific; when either PEA or SEA was combined with poly-L-lysine, there was negligible growth of *S*. *mutans* and biofilm formation. In contrast, OEA in combination with poly-L-lysine had a much stronger inhibitory effect on biofilm formation and then on bacterial growth, which is reflected in elevated growth/biofilm ratios of 3.5–6 (Table 1 (B)) at high concentrations of both agents and thus was considered as antibiofilm specific.

## 4. Discussion

*S*. *mutans* has been identified as the major bacteria responsible for initiating colonization of the oral cavity, and it is capable of generating an acidic environment that results in decalcification of the tooth [26]. Dental plaque control improved with the use of antiseptics, such as chlorhexidine and its derivatives, which affect oral bacteria and their attachment [27]. However, several side effects including odor, taste, aftertaste, alcohol, tooth staining, and low pH are associated with the use of the above formulations. Therefore, natural antimicrobials could provide potential alternative candidates to traditional mouthwash solutions in oral care [28, 29].

In the present study, we found that all tested agents when applied alone have minor to moderate activity against bacterial growth and biofilm formation. However, in mixtures of poly-L-lysine with either AEA or OEA, a strong synergistic antibacterial/antibiofilm effect was demonstrated. In contrast, two other tested EC-like agents, PEA and SEA, were not effective either alone or in combination with poly-L-lysine. Since all tested EC/EC-like have identical ethanolamine residue, this obvious difference may be attributed to their distinct fatty acid chain structure. Both AEA and OEA have double bonds in their fatty acid chain, while PEA and SEA have a saturated fatty acid chain. Indeed, it has been reported that the presence of double bonds in fatty acids plays an important role in its antimicrobial activity [30].

Biofilm formation is a major virulence determinant in *S*. *mutans* pathogenesis. Therefore, the prevention of biofilm formation plays an essential role in maintaining oral health. As opposed to killing (or arresting growth) of the microbe, which is what common antimicrobials do, specific antibiofilm agents are aimed at preventing biofilm formation without affecting pathogen viability.

We determined growth/biofilm formation ratios in order to evaluate the specificity of antibiofilm activity of the tested combinations. Mixtures of either PEA or SEA with poly-L-lysine were not effective against either biofilm formation or growth of *S*. *mutans*, which was reflected by a ratio of 1. On the other hand, the combination of AEA and poly-L-lysine was able to reduce growth and biofilm to the same degree, and, therefore, the calculated ratio was also equal to 1. Thus, the effect on biofilm formation of combinations of SEA, PEA, or AEA with poly-L-lysine was either negligible or nonspecific. In contrast, growth/biofilm ratios of OEA and polycation mixture at high doses were greater than 3, which indicate the specific antibiofilm effect of this combination. Indeed, the OEA and poly-L-lysine combination completely inhibited biofilm formation while allowing bacterial planktonic growth. Previously, we reported on the specific antibiofilm effect of different agents against fungi *C*. *albicans* [31] and bacteria MRSA [23].

The mechanism of synergistic activity could be explained by the fact that both EC/EC-like and poly-L-lysine may target the bacterial membrane. Poly-L-lysine with a high concentration of positive charges on the molecule exerts its antimicrobial activity by means of attachment onto the bacterial membrane via electrostatic force, followed by modification of the membrane properties [10]. Another study demonstrated that poly-L-lysine has the ability to decrease the content of large molecules, cellular soluble proteins, and nucleic acids associated with increasing the content of cytoplasmic *β*-galactosidase in the supernatant by causing damage to the cell membranes [11]. It is important to note that administration of poly-L-lysine either alone or in combination affects pathogenic bacteria without harming human healthy microflora [16, 17].

ECs are amphiphilic molecules that were demonstrated to have a strong affinity to mammalian cell membranes due to a nonspecific receptor-independent mode of interaction [32]. Some studies have reported a profound capacity of EC agents to change eukaryotic cell membrane properties, such as lipid bilayer fluidity [33] and elasticity [32]. In regard to the above, we propose that EC could affect the bacterial cell membrane lipid bilayer through a similar mode of action. The fatty acid chain, which is compatible with the lipid bilayer of the bacteria, can interact further with the lipid membrane and subsequently disrupt the cytoplasmic membrane [34]. Our previous data demonstrated that EC, AEA, and EC-like AraS at subkilling concentrations were able to destabilize the cytoplasmic membrane of MRSA [23]. The enhanced antibacterial effect of poly-L-lysine either in mixture [35] or conjugated to fatty acid [36] as compared to single treatment of each compound was documented. Furthermore, amphiphile-based poly-L-lysine demonstrated an obvious antibacterial effect against both Gram-positive and Gram-negative bacteria through electrostatic and hydrophobic interaction [37]. Previously, we postulated that mixtures of EC, AEA, or EC-like AraS compounds with common antibiotics exert antimicrobial activity towards MRSA through nonspecific interaction with bacterial membrane. We assumed that this membrane-targeting activity of AEA/AraS increases the uptake and antibacterial efficiency of antibiotic agent (unpublished data). Interestingly, introduction of hydrophobic groups onto poly-L-lysine enables solubility in an aqueous phase as well as allows the amphiphile to pass through highly lipophilic bacterial membranes [37]. Similarly, EC/EC-like and poly-L-lysine in combination could enhance permeability and efficiency of each other via hydrophobic and electrostatic interactions with *S*. *mutans* membrane.

This combination appears to be safe for human consumption, since mammalian cell membranes contain much less negatively charged lipids as compared to bacterial membranes. Thus, due to electrostatic attraction, cationic and amphiphilic molecules preferentially target bacteria, resulting in the selectivity of bacteria over human cells [38]. Based on this observation, the structure and function of antimicrobial peptides have been utilized as the basis for the discovery of cationic and amphiphilic molecules as potential and safe antimicrobial agents [39].

In conclusion, we assume that a combination of endogenous natural compounds such as EC/EC-like and poly-L-lysine may benefit oral hygiene by preventing dental plaque.

## Figures and Tables

**Table 1:** Growth/biofilm ratio.

Poly-L-lysine, *µ*g/ml	0	3.12	6.25	12.5	25
(A) AEA, *µ*g/ml					
0	1.00	1.01	0.99	1.17	1.19
1.56	0.90	0.93	0.96	0.99	0.97
3.12	0.97	0.95	0.93	1.00	1.03
6.25	0.91	0.94	0.86	0.92	0.96
12.5	0.92	0.93	0.92	1.07	1.02
25	1.00	0.97	0.78	0.54	1.16

(B) OEA, *µ*g/ml					
0	1.00	0.79	0.70	0.61	0.63
1.56	0.96	0.76	0.64	0.59	0.59
3.12	0.98	0.73	0.66	0.56	0.50
6.25	1.01	0.62	0.35	0.56	0.76
12.5	0.96	0.28	3.51	4.77	4.85
25	1.09	3.42	4.91	3.41	5.92

(C) PEA, *µ*g/ml					
0	1	0.968466624096462	1.00260000358007	1.02154390748878	1.02148518874135
1.56	1.00	0.95	0.88	0.90	1.01
3.12	1.03	0.98	0.98	0.97	0.98
6.25	1.04	1.00	0.98	0.97	1.10
12.5	1.03	1.13	1.20	1.23	1.17
25	1.02	1.24	1.36	1.31	1.21

(D) SEA, *µ*g/ml					
0	1	1.05982379672316	1.04938930850655	1.09277667421581	1.05733205582216
1.56	1.09	1.06	1.03	1.00	1.03
3.12	1.08	1.04	1.04	1.01	1.04
6.25	1.04	1.04	1.06	1.03	1.03
12.5	1.00	1.00	0.98	0.99	0.99
25	1.06	1.05	1.02	0.99	1.08

## Data Availability

The data used to support the findings of this study are available from the corresponding author upon request.
